# Development of Molecular Dynamics and Research Progress in the Study of Slag

**DOI:** 10.3390/ma16155373

**Published:** 2023-07-31

**Authors:** Chaogang Zhou, Jinyue Li, Shuhuan Wang, Jingjing Zhao, Liqun Ai, Qinggong Chen, Qiya Chen, Dingguo Zhao

**Affiliations:** 1 College of Metallurgy and Energy, North China University of Science and Technology, Tangshan 063210, China; zhouchaogang9@163.com (C.Z.); lijinyue2022@163.com (J.L.); ailiqun@ncst.edu.cn (L.A.); chenqinggong8@163.com (Q.C.); chenqiya@hotmail.com (Q.C.); zhaodingguo@ncst.edu.cn (D.Z.); 2 Tangshan Special Metallurgy and Material Preparation Laboratory, Tangshan 063210, China; 3 College of Pharmaceutical Sciences, North China University of Science and Technology, Tangshan 063210, China; zhaojingjing@ncst.edu.cn

**Keywords:** molecular dynamics, microstructure, slag, phosphate, silicate, aluminosilicate

## Abstract

Molecular dynamics is a method of studying microstructure and properties by calculating and simulating the movement and interaction of molecules. The molecular dynamics simulation method has become an important method for studying the structural and dynamic characteristics of slag systems and can make up for the shortcomings of existing detection methods and experiments. Firstly, this paper analyzes the development process and application fields of molecular dynamics, summarizes the general simulation steps and software algorithms of molecular dynamics simulation methods, and discusses the advantages and disadvantages of the algorithms and the common functions of the software. Secondly, the research status and application progress of molecular dynamics simulation methods in the study of phosphate, silicate, aluminate and aluminosilicate are introduced. On this basis, a method of combining molecular dynamics simulation with laboratory experiments is proposed, which will help obtain more accurate simulation results. This review provides theoretical guidance and a technical framework for the effective analysis of the microstructure of different slag systems via molecular dynamics, so as to finally meet the needs of iron and steel enterprises in producing high-quality steel grades.

## 1. Introduction

With the continuous development and maturity of modern technology, computer technology has been advancing by leaps and bounds; various research applications of molecular dynamics in metallurgical engineering have gradually increased, and the importance of molecular dynamics has become increasingly prominent [1,2,3]. Molecular dynamics in metallurgy can simulate crack propagation, solid-state phase transition and liquefaction processes and calculate structural properties and transport properties [4]. In metallurgy, it is possible to simulate not only solids but also liquids and the process of changing from melt to glass [5]. Aluminosilicate systems have always been the focus of every industry, including coal gasification, steel manufacturing, glass and other pyrometallurgical industries [6,7]. Phosphate glass is widely used because of its properties. It has higher electrical conductivity and a higher thermal expansion coefficient than silicate glass. The main components of phosphate glasses are [PO_4_] tetrahedra, which are interconnected by bridging oxygens [8,9,10]. Achieving good results in the above applications is closely related to system microstructure. Therefore, it is very important to study the microstructure of a system.

At present, there are many methods for studying the microstructure of a slag system. For example, the detection methods mainly include X-ray diffraction, Raman spectroscopy and neutron diffraction [11,12,13]. However, there are some problems with these detection methods. Some detections cannot be performed at high temperatures, and some errors will inevitably occur during the detection process. It is difficult to directly use the above methods to measure the structure and properties of a slag system. However, relying solely on the aforementioned methods may not be sufficient to solve certain metallurgical microstructure problems [14]. Therefore, in order to overcome the limitations of traditional detection methods and gain a better understanding of the reaction mechanisms in metallurgy, the application of molecular dynamics simulations has been increasingly utilized. In recent years, molecular dynamics has been widely employed for structural analysis of various slag systems. The main advantages of molecular dynamics simulation can be summarized as follows: (1) The relevant structural properties and kinetic properties can be provided from the atomic point of view, and the coordination number, bridging oxygen and micro-element structural unit Q^n^, bond length and bond angle can be analyzed directly from the microstructure. Moreover, the Raman spectrum obtained via calculation can be compared with the Raman spectrum obtained via measurement to compensate for errors caused by high temperature and other factors. (2) Molecular dynamics simulation methods are not limited by test equipment and sample preparation. (3) It can obtain the particle motion law and particle trajectory information that cannot be obtained via experiments, and it can determine the properties between the microstructure and the macrostructure. At the atomic scale, Li et al. [15] established the relationship between the nanoscale and macroscopic mechanical properties of excess-sulfate phosphogypsum slag cement. The molecular dynamics method can accurately capture the details of molecular interaction and microstructure in slag, thus providing a deep understanding of slag properties. Slag is a complex mixture composed of many compounds. The molecular dynamics method can simulate many different types of molecules and ions and their interactions. This flexibility enables the molecular dynamics method to deal with various types of slag systems, from simple binary slag systems to multi-component slag systems. There are also some limitations in molecular dynamics simulation methods. Due to the limitation of computing resources, molecular dynamics simulation can only simulate relatively small systems. This means that the simulation may not cover large-scale systems such as macromolecules, large proteins or complex nanomaterials. Although molecular dynamics simulation can simulate the motion of atoms and molecules, it can usually only simulate the time scale from nanoseconds to microseconds. For dynamic processes with longer time scales, such as protein folding or phase transition, more complex methods or other simulation techniques are needed.

This paper firstly introduces the development situation, simulation process and application fields of molecular dynamics, then summarizes the application of molecular dynamics in different slag systems, and finally puts forward the prospect of the development of molecular dynamics simulation in metallurgy. Through the molecular dynamics simulation method, experimental phenomena can be interpreted, the microstructure and its reaction mechanism can be clarified, and finally the metallurgical slag system of high-quality steel can be obtained, which provides a theoretical basis and scientific basis.

## 2. General Situation Analysis of Molecular Dynamics

### 2.1. Development of Molecular Dynamics, Simulation Process, Common Algorithms and Boundary Conditions

#### 2.1.1. Development Profile Analysis

**Figure 1:**
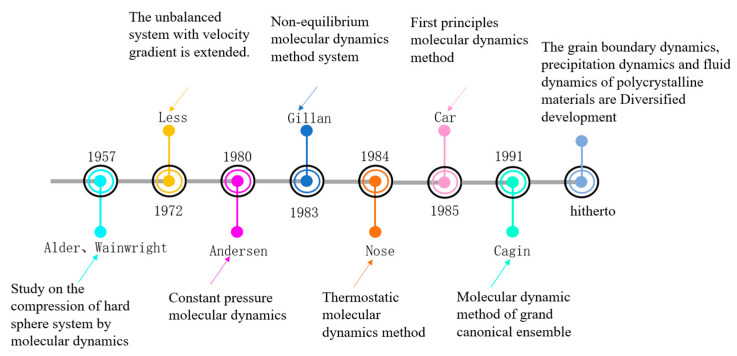
Overview of the development of molecular dynamics.

Molecular dynamics is the most widely used method in molecular mechanics. During the development of molecular dynamics, the molecular force field has been continuously improved. A large number of scholars have established force field systems suitable for metals, non-metals and polymers. These researchers laid a foundation for the development of molecular dynamics and the study of microstructure. Its development overview can be summarized as shown in Figure 1. It can be seen from Figure 1 that in 1957, in order to study the compression of hard sphere systems, Alder and Wainwright [16] used the molecular dynamics hard sphere model to simulate the conversion process between gas and liquid. This created a precedent for the use of molecular dynamics to study macroscopic properties. In 1972, Less et al. [17] further developed the Alder method and extended the non-equilibrium system with a velocity gradient to discuss the time-dependent properties and actions under extreme conditions, which has important practical and theoretical significance for the rupture of hydrodynamic behavior. In 1980, fluid was simulated at constant pressure and constant temperature, and Andersen et al. [18] created the constant pressure molecular dynamics method. In order to make molecular dynamics cover a wider field, cover more systems and have sufficient accuracy, in 1983, Gillan et al. extended this method to non-equilibrium systems with temperature gradients, and the molecular dynamics method for non-equilibrium systems was established. In 1984, Nose et al. completed the creation of the isothermal molecular dynamics method. In 1985, covalent bonding and metal system simulation was difficult, and Car et al. [19] put forward a first-principles molecular dynamics method to solve this problem by organically unifying electron theory and the molecular dynamics method. In 1991, Cagin et al. [20] further proposed the molecular dynamics method of a giant canonical ensemble to deal with adsorption problems. In recent years, molecular dynamics has developed many theoretical branches. Han et al. [21] relied on the disconnected description of grain boundary dynamics to summarize the concept of grain boundary dynamics for polycrystalline materials. Zheng et al. [22] conducted a study on the precipitation kinetics of FeCO_3_. Chen [23] combined molecular dynamics and computational fluid dynamics to study the pyrolysis of polymers.

Nowadays, molecular dynamics can not only obtain the trajectory information of particles but also observe it like an experiment and show a diversified development trend. It can be combined with other theories for research; in future development, we can conduct research towards more accurate and detailed microstructure simulations.

#### 2.1.2. Overview Analysis of Simulation Steps

**Figure 2:**
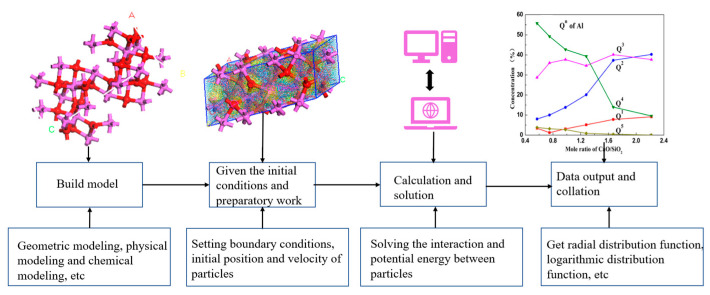
Molecular dynamics simulation process.

The general process of molecular dynamics simulation is shown in Figure 2. As shown in Figure 2, firstly, modeling is the first step in the simulation process, mainly including geometric modeling, physical modeling and chemical modeling [24,25]. Secondly, setting the initial conditions mainly includes setting the boundary conditions of the simulation area, setting the initial velocity and position of all particles and selecting the appropriate potential function. Commonly used potential functions include Lennard–Jones (L–J), BMH and EAM potential functions [26,27]. The appropriate potential function needs to be selected according to the state of matter. The full name of the BMH potential function is Born–Mayer–Huggins, which is a mathematical model describing the interaction between ions in ionic crystals and is widely used in silicate and aluminate systems. The EAM potential function is an embedded atomic potential, which can easily separate complex energy functions. Currently, it can predict and simulate the properties, structures, and mechanical behavior of various metals, non-metals, and composite materials. Thirdly, it is necessary to calculate the interaction force and potential energy between particles, as well as the position and velocity of particles. Static, dynamic, and quasi-static situations need to be considered in the calculation [28,29]. The final step is analysis, which involves sorting out the obtained results and connecting the macroscopic quantities such as temperature, volume, and pressure with the microscopic quantities [18,30], to obtain the radial distribution function (RDF), pair distribution function (PDF) and coordination number (CN), etc., thereby obtaining the changes in bond length, bond angle and polymerization degree.

#### 2.1.3. Overview Analysis of Common Algorithms

Most of the algorithms in molecular dynamics simulation are based on the finite difference method to integrate Newton’s equations of motion. The commonly used algorithms are as follows.

1. Verlet algorithm

The Verlet algorithm [31] was proposed in the late 1960s. Because the calculation process is relatively simple and the integration of the centroid motion of the diffusing molecules is the most stable, it is the most widely used in the simulation. It uses the position r(t) and acceleration a(t) of the atom at time T and the position r(t − δt) at time t − δ(t) to calculate the position r(t + δt) of the atom at time t + δ(t).

2. Speed Verlet algorithm

The speed Verlet algorithm [32] proposed by Swopo can give the position, velocity, and acceleration at the same time without consuming precision, which is an improved form of the Verlet algorithm.

3. Leapfrog algorithm

The leapfrog algorithm proposed by Hockney [33] is an algorithm derived from the Verlet algorithm. The leapfrog algorithm has two advantages over the Verlet algorithm: It does not need to calculate the difference between two larger quantities 2r(t) and r(t − δ), which reduces the amount of calculation, speeds up the calculation and reduces the error. But it also has one obvious disadvantage: The position coordinate r(t + δt) and the velocity cannot be synchronized; that is, after the position of each particle in the system is determined, the potential energy of the system can be determined, but the contribution of the kinetic energy of the system to the total energy at this moment cannot be calculated.

4. Beeman algorithm

The Beeman algorithm [34] is an algorithm proposed by Beeman Ullman, which is similar to the Verlet algorithm. Its advantage lies in its ability to refer to a longer integration interval, and the cited integration step size is 3–4 times that of the Verlet algorithm. It has the same accuracy, and it is a particularly suitable algorithm to describe the molecular dynamics of liquids and dense gases, and it is another widely used algorithm.

5. Rahman algorithm

The Rahman algorithm [35] is an integral method first used by Rahman, which is a variant of the prediction–correction algorithm. Although the Rahman algorithm can achieve a more accurate solution to the equation of motion, it has a large amount of calculation and takes a long time, so it is relatively less used now.

It can be seen from the above analysis and comparison that two sets of initial positions must be given when using the Verlet algorithm, so it is not a self-starting algorithm, but the calculation is simple and easy to implement, making it widely used. When using the Speed Verlet algorithm for the Verlet algorithm, the stability of numerical calculation is enhanced. The leapfrog algorithm sacrifices some accuracy compared to other algorithms, but it performs better in long-term molecular dynamics simulations. The Beeman algorithm is a variant of the Verlet algorithm; its storage capacity is larger than that of the leapfrog method, but the calculation amount is too large. Compared with other algorithms, the Rahman algorithm can obtain more accurate results, but it will take longer to calculate. Algorithms play a vital role in the study of molecular dynamics. Various algorithms are formed using different integration methods. Each algorithm has different formulas that make them different in terms of efficiency, advantages, and disadvantages. The above commonly used algorithms’ advantages and disadvantages are summarized in Table 1. According to Table 1, it can be seen that the Verlet algorithm and Leapfrog algorithm are relatively simple in terms of the calculation process, and the Speed Verlet algorithm and Rahman algorithm are relatively accurate in the calculation. However, the accuracy of the algorithm mentioned above will also be affected by other factors, such as the choice of the time step, the accuracy of the force field model, the size of the simulation system, and the boundary conditions. Therefore, in molecular dynamics simulation, it is necessary to comprehensively consider various factors and choose the algorithm suitable for the system.

#### 2.1.4. Boundary Conditions

**Figure 3:**
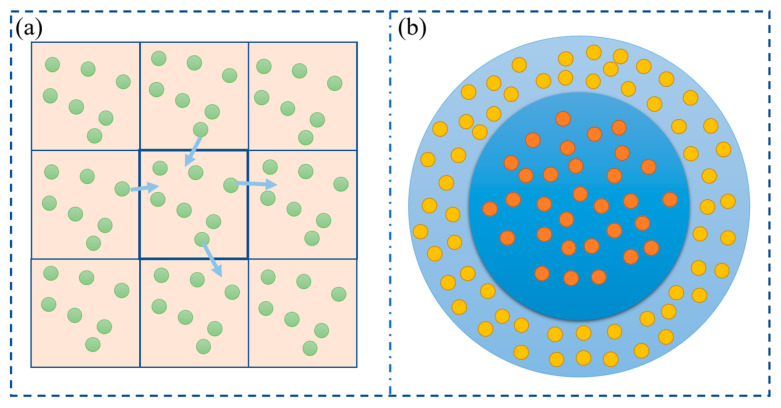
(**a**) Periodic boundary condition (The arrow is the direction of particle movement. If particles run out of the model from the right, particles will return to the model from the left.). (**b**) Aperiodic boundary condition.

At present, it is difficult for computers to directly deal with the molecular dynamics model of the actual system with more than one million ions, so the total number of particles must be controlled to make it balance at a fixed value. At present, the method adopted is periodic boundary conditions [36]. By introducing periodic boundary conditions, if one or several particles run out of the model, the same number of particles will return to the model from the opposite interface, thus achieving a constant number of particles in the system. The schematic diagram of the periodic boundary is shown in Figure 3a. Some systems need to use aperiodic boundary conditions, such as droplets or atomic clusters, which contain interfaces themselves. Another example is a non-uniform system or a system originating from non-equilibrium, then aperiodic boundary conditions need to be used. As shown in Figure 3b, the shell in the figure—that is, the shaded part—is the boundary area of the system. The molecules in the boundary area can be designed to be in a fixed position, and the molecules in the middle area can be moved.

### 2.2. General Analysis of Common Software for Molecular Dynamics Simulation

**Figure 4:**
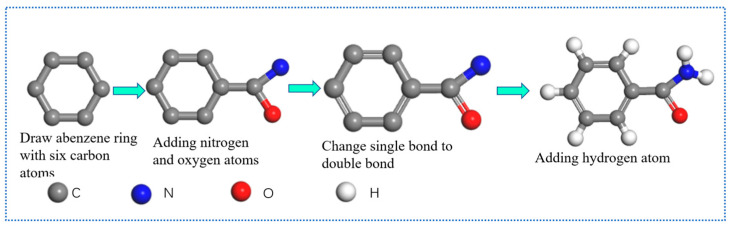
MS software to draw benzamide molecules.

Commonly used software for molecular dynamics simulation includes Lammps, Materials Explorer and ovito (version number: 3.4.4) software. Among them, Materials Explorer (version number: 4.0) is a multi-functional molecular dynamics software, with powerful molecular dynamics calculation and Monte Carlo software package, a perfect graphical interface and easy operation. Unlike Materials Explorer, Lammps is an open-source type, does not have a graphical interface, and performs calculations by writing code. Unlike the above software, ovito does not have a simulation function. It is a visualization and analysis software for particles and atoms. The Lammps [37] (version number: 27Oct2021-MPI) software is a classical molecular dynamics software. It uses different force fields and boundary conditions to model atomic, polymeric, biological, metallic, and granular systems. Lammps can calculate systems ranging from a few particles to millions or even hundreds of millions of particles. It has particularly rich support for particle types and force fields, the simulation object is not limited to a certain type of system, and it is widely used. Lammps can write its code to build a model and can also build a model through Materials Studio (MS, version number: 20.1.0.2728) and then convert it into the required data file and directly import it into Lammps for the next step. Materials Studio can easily create and study material structures or molecular models and has excellent modeling and drawing capabilities [38]. The process of drawing benzamide molecules using MS software is shown in Figure 4. It cannot only be applied to molecular dynamics; it can also be used in quantum mechanics and mesoscopic models. The interior of the software is modular, with each module providing a different approach to structure determination, property prediction or simulation. After the Lammps software calculation is completed, it can be analyzed with visualization software such as VMD (version number: 1.9.3) and AtomEye (version number: 3).

According to Figure 4, to draw a benzamide molecule, first, draw a benzene ring with six carbon atoms, then draw another carbon atom on the carbon atom on the benzene ring, and connect an oxygen atom and a carbon atom to the new carbon atom, then the three non-adjacent bonds and carbon–oxygen bonds on the benzene ring are changed from single bonds to double bonds; finally, hydrogen atoms are added, and the benzamide molecule is drawn.

Discover in Materials Studio is a molecular mechanics calculation engine, which can accurately calculate the lowest energy conformation and give the dynamic trajectories of the architectures under different ensembles. It also has powerful analysis tools, which can analyze the simulation results to obtain structural parameters and thermodynamic properties, etc. Polymorph Predictor is a set of algorithms that can determine low-energy polymorphs of crystals. This module can be combined with experimental data for analysis; similarity selection and a clustering algorithm are used to classify similar models to save computing time. CASTEP is a quantum mechanical program widely used in metals, semiconductors, and ceramics, among others. The properties, energy bands, density of states, extended defects and charge density of crystalline materials can be studied via CASTEP [39]. DPD is an advanced mesoscopic simulation method used to study complex fluid phenomena, including pigments, pharmaceuticals, and cosmetics. The QSAR module is a comprehensive toolset that can compute descriptors of molecules and make connections between descriptors and properties and can also predict the activity of unknown materials.

**Figure 5:**
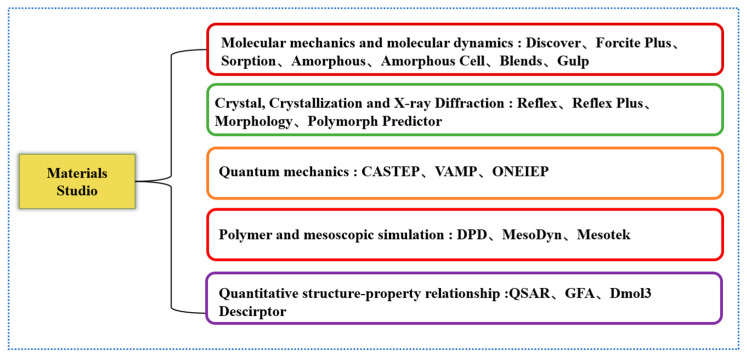
MS module introduction.

To sum up, in terms of nanomaterials, property detection, and relatively complex quantum mechanics and molecular dynamics simulations, the simulation results can be analyzed to obtain the required data and solve some key problems in current metallurgy and materials. MS can perform computational simulations on problems in different fields through different modules, and a summary of each module included in it is shown in Figure 5.

### 2.3. Overview of Molecular Dynamics Application Fields

Molecular dynamics can directly analyze the bond lengths, bond angles, coordination relationships, types of oxygen ions, and types of microscopic structural units of different particle pairs from a microscopic perspective. Through the numerical simulation and statistical summation of the microstructure and motion process of matter, the law of particle motion and the relationship between the microstructure and the macrostructure can be obtained [40,41]. Molecular dynamics adopts the classical Newtonian mechanics model, so it can only describe the nuclear motion, and cannot reflect the electron motion [42].

**Figure 6:**
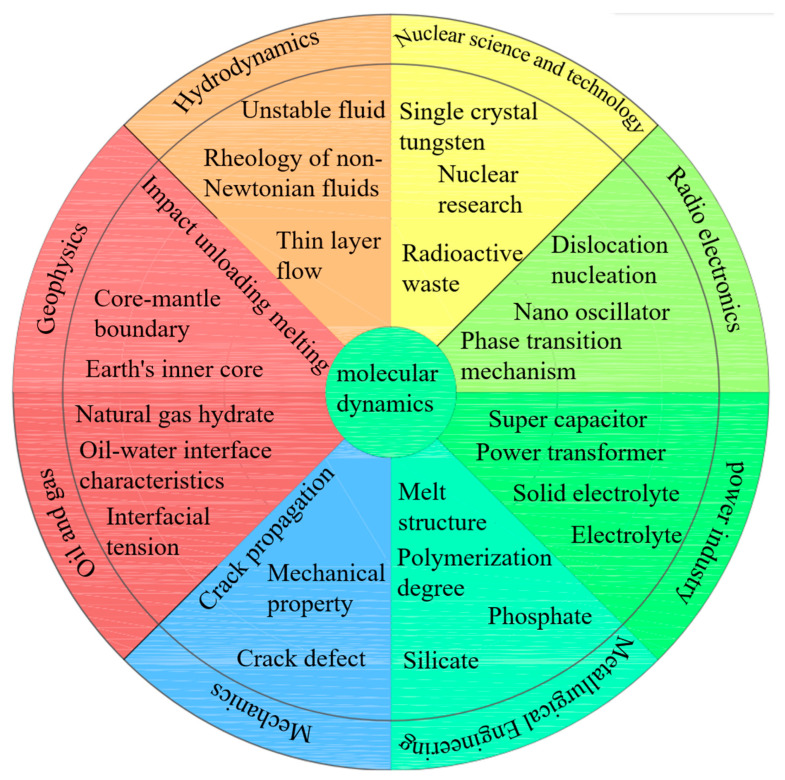
Molecular dynamics application areas.

Molecular simulation refers to the use of theoretical methods and computational techniques to simulate or simulate the microscopic behavior of molecular motion, and is widely used in computational chemistry, computational biology, and materials science [43,44]. The object of its research can be as small as a single chemical molecule or as large as a complex biological system or material system [45,46,47]. Molecular dynamics also has some applications in the field of ultra-precision machining [48,49]. In the current hot electrolyte field, there are frequent reports on molecular dynamics simulations [50,51,52,53]. Due to experimental limitations and the fact that the macroscopic properties of slag are determined by its microstructure, molecular dynamics simulations have been widely used in the structural analysis of metallurgical slag in recent years [54,55,56]. According to the previous research on the molecular dynamics research field, it can be summarized as shown in Figure 6. It can be seen from Figure 6 that molecular dynamics is mainly used in metallurgical engineering, fluid mechanics [57], nuclear science and technology [58], oil and gas engineering [59], and mechanics. In metallurgical engineering, mainly the degree of polymerization and structure are analyzed. In terms of mechanics, the defects, propagation and mechanical properties of cracks can be studied [60,61,62], and electrolytes and transformers can be studied in electric power [63,64]. In the application of fluid dynamics, the rheology of non-Newtonian fluid can be simulated, and thin-layer flow and unsteady fluid can also be simulated. Molecular dynamics simulations can be used to study the thermal conductivity of radioactive waste and tungsten in nuclear science and technology. Molecular dynamics can be used to study the adsorption and diffusion process of shale oil and the characteristics of the oil–water interface, and to explain the experimental phenomena related to oil production, which can improve the oil recovery rate. In radio electronics, subsurface damage, a phase change mechanism, and a nano oscillator can be simulated. For geophysics, molecular dynamics helps study the state and properties of matter in the Earth’s interior environment.

**Figure 7:**
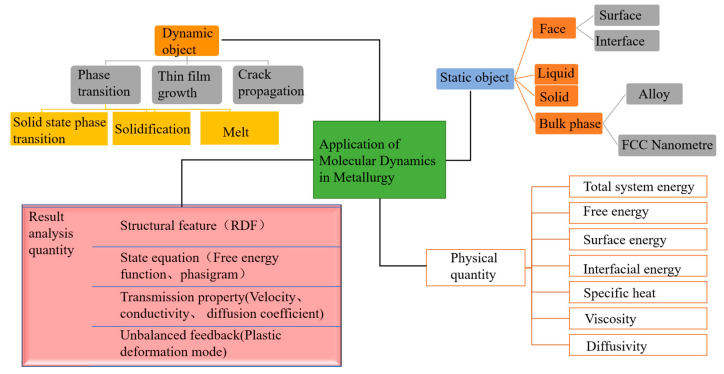
Application of molecular dynamics in metallurgy.

The application of molecular dynamics in metallurgy is shown in Figure 7. In metallurgy, molecular dynamics can study the relationship between the structure and properties of slag, the structure of metal melt and the properties of nano-scale inclusions, and can obtain information such as the material structure, the movement law of ions, and the relationship between microstructure and macro-structure by simulating and statistically summing the microscopic material and movement process [65]. It can also analyze solids and liquids, metal–metal interfaces, solid–liquid interfaces, and liquid–gas interfaces [66,67,68,69]. It is also able to understand phase transitions, deposition and crack growth of dynamic objects [70,71]. The static structure factor can be obtained to determine the degree of structural disorder. Specifically, the statistical fluctuations of the mean square displacement MSD, coordination number, particle trajectory, and kinetic energy can be analyzed [72,73].

## 3. Research and Application Analysis of Molecular Dynamics in Slag

### 3.1. Research Status and Application Analysis in Phosphate Residue

Phosphorus is one of the most harmful elements in steel, and steel with high phosphorus content is prone to cracking, reducing the corrosion resistance and toughness of steel, etc. Therefore, it is very important to remove phosphorus from molten iron with high phosphorus content [74,75,76,77]. The phosphate melt is a key part of the dephosphorization slag; a detailed study of its microstructure is helpful to deeply understand the properties of slag. There have been many studies on phosphate compounds involving molecular dynamics in recent years. Phosphate glasses have unique crystallization, chemical corrosion resistance, and other properties, as well as a low scattering rate, high refractive index, and high thermal expansion coefficient, which are widely used in sealing materials, optical components, solid electrolytes, biomedical glasses, and so on. Phosphate-based glasses also have overlapping applications in the fields of optics and information science, including auto-driving, machine vision, and virtual reality technology. Phosphate compounds also have many applications and research in cement [78,79].

**Figure 8:**
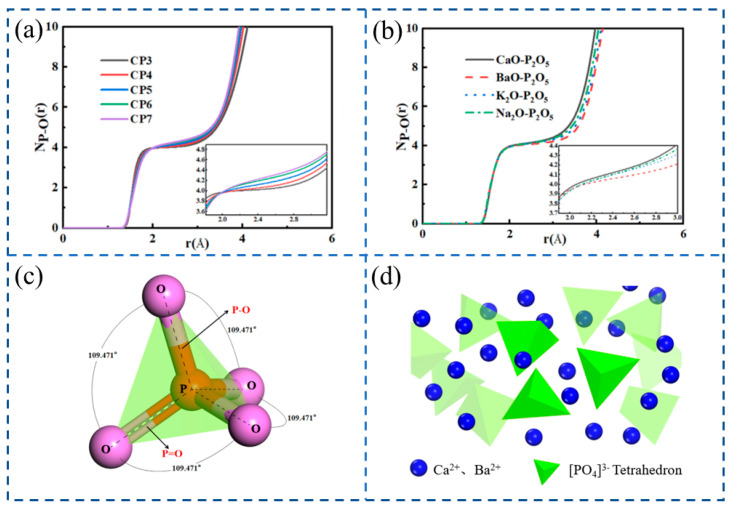
(**a**) The CNs of the P-O in the CaO-P_2_O_5_ binary system with varying P_2_O_5_ contents. (**b**) The CNs of the P-O in different P_2_O_5_-based binary systems when P_2_O_5_ is 50 mol%. (**c**) Schematic diagram of the standard tetrahedron. (**d**) The structure of phosphate melts [80].

Du et al. [80] studied the microstructural characteristics and evolution laws of different P_2_O_5_-based binary melt structures; as can be seen from Figure 8a, with the increase in P_2_O_5_ content, the CNs platform slightly inclines upward, which means that the average coordination number of P increases. It can be seen from Figure 8b that when the content of P_2_O_5_ is 50 mol%, the order of CNs in different P_2_O_5_ binary systems is BaO-P_2_O_5_ < K_2_O-P_2_O_5_ < Na_2_O-P_2_O_5_ < Cao-P_2_O_5_, which means that the influence on the structural stability of P-O is BaO < K_2_O < Na_2_O < CaO. And it can be observed that the ordinate of the CNs curve of P-O is slightly larger than four, indicating that most P atoms have a four-coordinated structure with oxygen. The study also shows that the basic unit of the P_2_O_5_-based binary melt network structure is [PO_4_]^3−^, and the structure is close to the standard tetrahedron, as shown in Figure 8c. And the local structure of phosphate melt is predicted as shown in Figure 8d, with green representing [PO_4_]^3−^ tetrahedron and blue representing Ca^2+^, Ba^2+^ and other metal cations. The whole system is composed of a large number of [PO_4_]^3−^ tetrahedrons, and metal cations will be dispersed in the gaps of the tetrahedrons, which can be used as modification units of the network structure to depolymerize the complex network structure.

**Figure 9:**
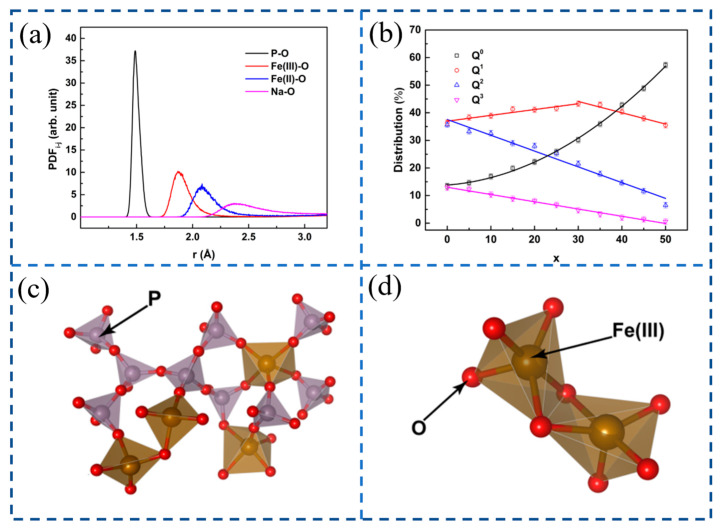
(**a**) PDF function. (**b**) Distribution of Qn structural units. (**c**) Angle connection. (**d**) Edge connection [85].

Phosphate systems are not only one of the main components of steel slag in the iron and steel industry, but also play an important role in other industrial processes such as chemical and glass industries [81]. Due to the low utilization rate of steel slag, which is only about 30%, and the concept of a green circular economy, recycling steel slag into the iron and steel making process is considered to be the most efficient way to utilize a large amount of steel slag [82]. Wang et al. [83] studied the structure of the CaO-SiO_2_-P_2_O_5_ ternary system. The results show that P_2_O_5_, as an acidic oxide, has a greater affinity for Ca^2+^ ions, capable of removing Ca^2+^ ions combined with Si-NBO (non-bridging oxygen), thereby forming the Si-O-Si bond and Ca-O-P bond. The structure of 2ZnO·P_2_O_5_-2Na_2_O·P_2_O_5_ was studied by Boiko et al. [84] Studies have shown that a distribution of building blocks is found in the anionic phosphate structure, including orthophosphate, pyrophosphate, and longer metaphosphate chains. Phosphate glass can be used in many fields such as medicine, optics, electronics, environmental protection, and so on because of its properties. Unfortunately, the chemical durability of pure phosphate glass is low, limiting its application. Goj et al. [85] studied the effect of Na_2_O addition on glass. As can be seen from Figure 9a, the image has a broad Na-O peak of PDF function, so sodium is a glass network modifier. Iron may play a dual role in the glass network. The first peak of the PDF function of Fe (II)-O and Fe (III)-O is wider than that of P-O, and it can be a glass network former or modifier. The research also shows that with the increase in Na_2_O content, Q^2^ units are transformed into Q^1^, Q^3^ units are gradually transformed into Q^0^ and the [PO_4_] tetrahedron is separated. When Na_2_O content exceeds 30mol%, the number of Q^1^ units begins to decrease, thus realizing the depolymerization of the glass network; the variation of Q^n^ is shown in Figure 9b. At the same time, it is found that the iron polyhedron can be connected by corners and edges, which realizes the continuity of the glass network; schematic diagrams of the corner connection and edge connection are shown in Figure 9c,d.

Rao et al. [86] investigated the effect of adding LiCl to dibasic phosphate glasses. The results show that the addition of LiCl in glass will produce more non-bridging oxygen and reduce the length of P-O-P chains in these chains, resulting in the weakening of the glass matrix. At the same time, with the addition of Cl, the O-Li-Cl interaction increases, creating better channels for Li^+^ movement. Ainsworth et al. [87] carried out a molecular dynamics simulation on phosphate group P_2_O_5_-CaO-Na_2_O; the simulation results show that phosphorus atoms are mainly combined with two or three oxygen atoms, and these oxygen atoms are connected with adjacent phosphorus atoms. Among all the components studied, sodium ion and calcium ion regulators occupy a pseudo-octahedral bonding environment, and the average oxygen coordination numbers are 6.55 and 6.85, respectively. Phosphate glass has become the most widely used laser glass medium because of its high solubility of rare earth ions, good spectral properties of rare earth ions, and low nonlinear coefficient; P_2_O_5_ can improve the dispersion coefficient and ultraviolet transmittance of glass.

Most of the above studies are about basic structure. For example, adding Na_2_O to the glass containing P_2_O_5_ will lead to the depolymerization of the glass network, increasing the amount of non-bridging oxygen in the tetrahedron, adding LiCl will also produce more non-bridging oxygen, and lead to the weakening of glass matrix. Besides the research on structure, the diffusion behavior of different atoms should also be investigated, such as the distribution difference of atomic density at different times and different diffusion distances. Most of the ensembles used by the above scholars are canonical ensembles (NVT), which are suitable for systems that can exchange energy with large heat sources and have a fixed number of particles in which the number of particles does not change to N, the volume does not change to V, and the temperature does not change to T. The study of molecular dynamics in phosphate and the selection of potential function is shown in Table 2. According to Table 2, most of the potential functions used in molecular dynamics simulation in phosphate research are BMH potential functions, among which Goj and Ainsworth choose a combination of multiple potential functions for simulation. The combination of potential functions can better describe the complexity and diversity of the system, improve the calculation efficiency and reduce the calculation time.

### 3.2. Research Status and Application Analysis in Silicate Slag

Silicate slag refers to the general term for compounds formed by combining silicon, oxygen, and other chemical elements (mainly aluminum, iron, calcium, magnesium, potassium, sodium, etc.). Silicate slag can be divided into three types: independent tetrahedral structure, polytetrahedral ring structure, and chain structure. Silicate slag products and materials are widely used in various industries, scientific research, and daily life. They can also be used as silicate coagulants to treat urban sewage and industrial wastewater, and their applications in water treatment are increasing. In the construction industry, Portland cement is widely used because of its good frost resistance, good wear resistance, and high hydration heat. Wu et al. [88] studied the microstructure and transport properties of the CaO-SiO_2_ system and discussed the relationship between structure and transport properties. By statistically calculating the diffusivity of oxygen ions, the relationship between the viscosity and electrical conductivity of the CaO-SiO_2_ binary system and the microstructure was obtained. And it is shown that with the increase in CaO content, the melt structure will be depolymerized, resulting in a decrease in viscosity and enhanced conductivity of the system. Yao et al. [89] studied the effect of CaF_2_ on the structure of the CaO-SiO_2_-CaF_2_ ternary slag system. The results show that CaF_2_ is mainly used as a diluent to fill the gaps in the network structure, and the rest partially reduces the polymerization degree of slag. Matsumiya et al. [90] studied the CaO-SiO_2_-CaF_2_ ternary system and showed that CaF_2_ does not affect the bond length between the bonding particle pairs in the system. Three potential functions, BMH, Buckingham and MiTra, are used in this study. Asada et al. [91] conducted a study on CaO-SiO_2_-CaF_2_ and concluded that when the CaF_2_ concentration is low, the polymerization of silicates is affected by the basicity of the slag and is hardly affected by F ions. They also compared the self-diffusion coefficients between ions: D_F_ > D_Ca_ > D_O_ > D_Si_. Fan et al. [92] simulated the structure of slag systems with different P_2_O_5_/SiO_2_ ratios at a fixed CaO content by molecular dynamics in the CaO-P_2_O_5_-SiO_2_ ternary slag system. The potential function used in this study is Born–Mayer–Huggins (BMH). And in this study, it is pointed out that the average bond length of Si-O is 1.610 Å and that of P-O is 1.531 Å. With the increase in P_2_O_5_ concentration, the bond length of Si-O does not change, which indicates that Si-O is not sensitive to the change of P_2_O_5_; the bond length of P-O decreases with the increase in P_2_O_5_ concentration, which indicates that the binding of P-O is tighter. The O-O bond length will decrease, and the oxygen atoms in the PO_4_ tetrahedron will increase significantly. The above phenomenon also occurs in CaO-SiO_2_-Al_2_O_3_ ternary slag systems with different Al_2_O_3_/SiO_2_ ratios. Diao et al. [93] studied the transport properties of molten CaO-SiO_2_-P_2_O_5_-FeO systems. Studies have shown that the diffusivity of Ca, Si, P, Fe, and O ions increases with the increase in slag basicity and FeO content, but decreases with the increase in P_2_O_5_ content; the diffusivity in this system is Ca > Fe > O >P > Si. Liang [94] analyzed the coordination structure of the CaO-B_2_O_3_-SiO_2_-TiO_2_ quaternary slag system; in addition, the effect of TiO_2_ content on the coordination structure of mold flux was studied. The results show that Ti and O in the quaternary slag system mainly exists in the form of a six-coordinated octahedral structure; the coordination structure of B has only four-coordinated tetrahedra and a three-coordinated triangular body. With the increase in TiO_2_ content, the coordination forms of Si and Ti in the microstructure are transformed into tetra- and penta-coordinate structures, respectively.

The above studies show that, in the silicate containing CaF_2_, when the concentration of CaF_2_ is not high, the change in the basicity of the slag will affect the polymerization reaction and viscosity, and the effect of F ions is not large. When CaF_2_ reaches 20–30 mol%, the influence of F ions increases gradually. F does not participate in the formation of the network structure and will combine with Ca and dissociate in the network gap, reducing the melt viscosity. Among them, only Matsumiya used the three potential functions of BMH + Buckingham + MiTra; the rest all use the BMH potential function. Matsumiya used the Buckingham potential function to study Ca-F and the MiTra potential function to analyze Si-F and F-O and was the first person to use multiple potential functions. Using different potential functions for different ionic bonds will have higher accuracy compared to other single potential functions, and more potential functions can be developed for application to different studies in the future. The research on the molecular dynamics simulation in the silicate slag system and the selection of the potential function are summarized in Table 3. According to Table 3, it can be seen that the ensemble used in the study of molecular dynamics simulation in silicate slag system is the NVT ensemble, and most of the components studied include CaO-SiO_2_-CaF_2_ or CaO-P_2_O_5_-SiO_2_.

### 3.3. Research Status and Application Analysis in Aluminate Slag

**Figure 10:**
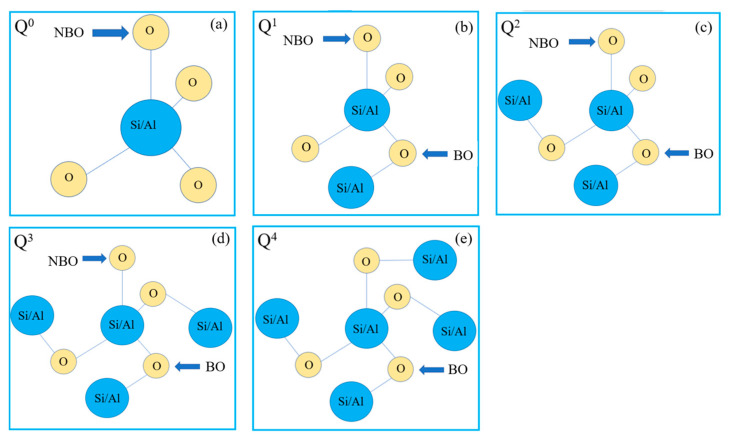
Schematic diagram of the microstructural unit.

In aluminate slag, Al_2_O_3_ is an amphoteric oxide with a complex structure. As the basic oxide increases to a certain extent, it leads to an increase in the degree of polymerization, which results in an increase in viscosity and a decrease in conductivity. When all Al^3+^ rows form [AlO_4_]^5−^ tetrahedrons, the melt will depolymerize, at which point the viscosity will decrease and the electrical conductivity will increase. Aluminate cement has obvious characteristics of quick hardening, low alkalinity, slight expansion, and good frost resistance, which can be widely used in winter construction, rapid construction, and the repair of marine and underground special projects. Aluminate materials can also be used as luminous materials for billboards and safety signs and are also widely used in ink, plastics, fibers and coatings. Xiao et al. [95] studied the binary system SiO_2_-Al_2_O_3_, and the results show that all Si in the system exists in the system in the stable structure of Si-O tetrahedron. Al mainly forms an Al-O tetrahedral structure in the system, and the stability of the single Al-O tetrahedral structure is lower than that of the Si-O tetrahedral structure. The increase in Al_2_O_3_ content leads to a slow increase in the average Al-O bond length. The radial distribution function and coordination number, bridging oxygen, and micro-element structural unit Q^n^ are also analyzed. Q^n^ includes five micro-element structural units, namely Q^0^, Q^1^, Q^2^, Q^3^, and Q^4^. Q^n^ is used to express the complexity of the slag network structure, where Q expresses the silicon–oxygen or aluminum–oxygen tetrahedral structure, and n is the number of bridging oxygens in the tetrahedral structure formed by the grid, a schematic diagram is shown in Figure 10. Figure 10a–e show a monomer Q^0^, a dimer Q^1^, a chain structure Q^2^, a ring structure Q^3^, and a fully polymerized unit Q^4^, respectively.

**Figure 11:**
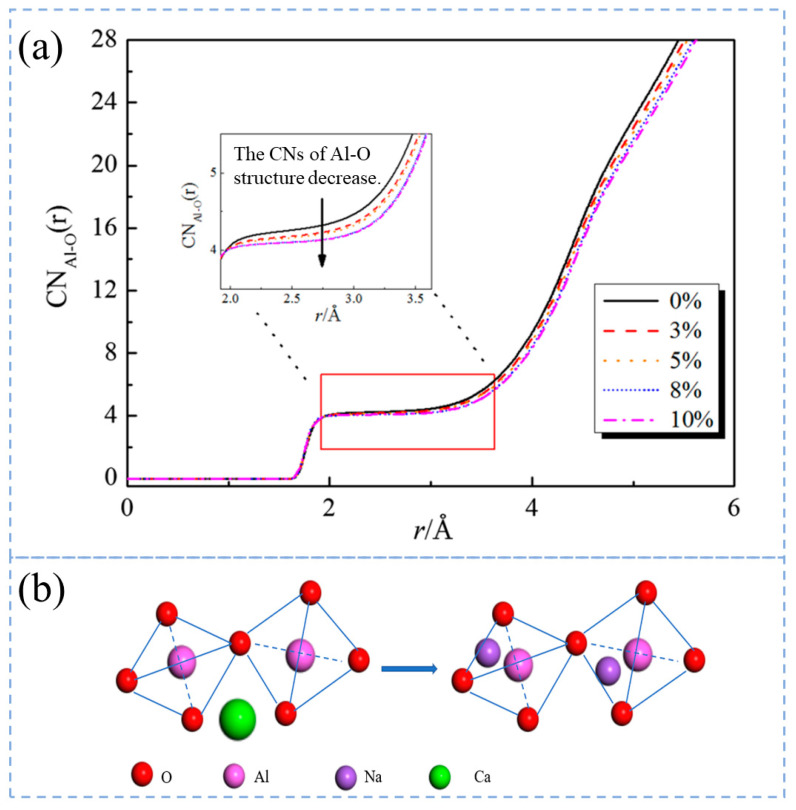
(**a**) CNs of Al–O with different contents of Na_2_O. (**b**) Schematic diagram of Na^+^ substitution for Ca^2+^ [97].

In the CaO-Al_2_O_3_ system, Wu et al. [96] studied the coordination number of Al and microstructural unit distribution, and the potential function used was BMH potential. The conclusion is that the microstructure unit distribution of calcium aluminate melt changes with the macro-composition, and the relationship between viscosity, conductivity, and slag polymerization degree is obtained. Zhang et al. [97] studied the replacement of Na^+^ by Ca^2+^ in the ternary slag system CaO-Al_2_O_3_-Na_2_O, Ca^2+^ mainly plays the role of modifying the network, and Na^+^ mainly plays the role of charge compensation of AlO_4_ tetrahedron. With the substitution of Ca^2+^ in slag by Na^+^, the charge compensation ability in the solution is enhanced, and the network modification ability is weakened. Some weak non-bridging oxygen in the form of Al-NBO-Ca is transformed into strong bridging oxygen in the form of Al-BO-Al. It can be seen from Figure 11a that with the increase in Na_2_O content, more Na^+^ will replace Ca^2+^ in CaO-Al_2_O_3_-Na_2_O melt, and the CNs of Al-O structure will decrease, and the spatial configuration will become more reasonable, as the CNs of Al-O structure tends to have four coordinates. The schematic diagram of Na^+^ replacing Ca^2+^ is shown in Figure 11b. By replacing Ca^2+^ with Na^+^, the geometrical difficulties required for the charge balance of two AlO_4_ tetrahedra through the same Ca^2+^ in the melt are eliminated, and the replacement of Ca^2+^ ions with Na^+^ ions is beneficial to the stabilization of the Al–O tetrahedral structure by the CaO-Al_2_O_3_-Na_2_O melt.

Zhang et al. [98] studied the changes in the short-range structure, medium-range structure, and bond angle of the slag system with different contents of CaF_2_ in the ternary slag system CaO-Al_2_O_3_-CaF_2_. The results showed that with the addition of CaF_2_, there is a dynamic equilibrium between Ca^2+^ and the coordinated anions, and the total coordination number is maintained between 6 and 7. The Al-O tetrahedral structure is transformed from Q^4^ and Q^3^ to Q^2^ and Q^1^, and the Al-O tetrahedral [AlO_4_]^5−^ will transform to the [AlO_3_F]^4−^ structure.

Through the above analysis, adding Na_2_O to aluminate, Na^+^ will gradually replace Ca^2+^ and will make the structure more stable. F has a dual effect on the melt structure. On the one hand, F will replace the O^2−^ position coordinated with Ca^2+^ to form Ca-F, which will dilute and reduce the viscosity. On the other hand, F reacts with [AlO_4_]^5−^ to obtain [AlO_3_F]^4−^ tetrahedral structure. The mechanism of action of Na^+^ and F^−^ on aluminate and the influence on its structure were successfully described, which provided theoretical support for future research on the reaction mechanism in aluminate. The potential functions used in the above research are Buckingham and BMH. The Buckingham potential function describes the repulsion principle and Van der Waals energy of the interaction between two atoms that are not directly bonded according to the distance R between atoms, the research of molecular dynamics in aluminate slag system and the selection of potential function are summarized in Table 4. According to Table 4, the ensemble selected by molecular dynamics in the aluminate slag system is the NVT ensemble, and the potential functions are BMH and Buckingham. Buckingham’s potential function is a commonly used empirical potential function, which is used to describe the interaction between atoms, but there may be some errors, and the parameterization process is difficult. Moreover, the performance of Buckingham’s potential function is highly dependent on the selected parameters, and different parameters may lead to different simulation results.

In the research, it is suggested to compare various potential functions to determine the best model structure. By applying different potential functions, we can obtain more comprehensive results and find the most suitable potential function for the research object. Such a comprehensive comparison can help researchers to deeply understand the advantages and limitations of the model, to obtain more comprehensive and accurate results. Yin et al. [99] studied the influence of different potential functions on the simulation results, showed that different potential functions lead to different microstructure characteristics and mechanical properties, and selected the potential function most suitable for mechanical deformation. On this basis, the effect of strain rate on mechanical deformation was studied. However, several aspects should be paid attention to when comparing various potential functions. First of all, the selected potential function must be widely applicable and reliable. Secondly, the comparative experimental conditions should be representative and should be carried out on appropriate time and energy scales. Finally, the comparison results need to be statistically analyzed and verified to ensure the reliability and repeatability of the conclusions.

To sum up, by comparing various potential functions, researchers can evaluate the performance and applicability of the model more comprehensively and provide more accurate and reliable results for research. This method can not only help researchers better understand the advantages and limitations of the model, but also promote the further development and innovation of scientific research in the field of molecular dynamics simulation.

### 3.4. Research Status and Application Analysis in Aluminosilicate Slag

**Figure 12:**
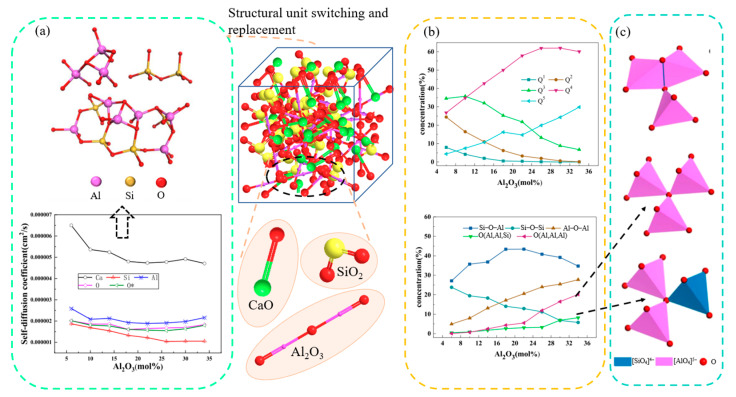
(**a**) Self-diffusion coefficient. (**b**) Qn and structural changes. (**c**) O (Al, Al, Si) and O (Al, Al, Al) structure.

The network structure of aluminosilicates consists of tetrahedra (SiO_4_ and AlO_4_) connected by bridging oxygens. Bridging oxygen is the oxygen that connects two tetrahedra, plays a connecting role, and plays a very important role in the aluminosilicate network structure; the oxygen content of the bridge directly affects the complexity of the system. The non-bridging oxygen is the opposite of the bridging oxygen; the oxygen ion that is not shared by the two polyhedra is the non-bridging oxygen, which can express the fracture degree of the network. Free oxygen is the oxygen ion that exists in the slag in the form of O^2−^. The aluminosilicate system can be used to make glass and ceramics, and can also be used as a pigment for glass and a filler for paint, rubber and plastic, and it is also applied in the medical field. Nano-aluminosilicate vaccine adjuvant has excellent adjuvant performance and can reduce aluminum accumulation in vivo. Feng et al. [100] used molecular dynamics to study the microstructure and thermal conductivity of the ternary slag system CaO-Al_2_O_3_-SiO_2_. The potential function used is BMH potential, and mainly the radial distribution function, oxygen type, and microstructure unit Q^n^ are studied. This study shows that both silicon and aluminum exist in the slag with a tetrahedral network structure, and the silicon–oxygen tetrahedron is more stable. The increase in Al content leads to a decrease in the bond angles of Al-O-Al and Si-O-Al, and the increase in BO and TO content in the network. Chen et al. [101] studied the effect of Al_2_O_3_ on the structure and dynamic properties of CaO-SiO_2_-Al_2_O_3_ melt through molecular dynamics simulation. The analysis summary is shown in Figure 12. As can be seen from Figure 12a, the self-diffusion coefficient of atoms in the system decreases in the order of D_Ca_ > D_Al_ > D_O_ > D_Si_. The value of D_O_ is similar to that of D_Si_ and D_Al_, which is between D_Si_ and D_Al_. With the increase in Al_2_O_3_ content, D_O_ is closer to D_Al_, so O atoms move with Al and Si atoms. It can be seen from Figure 12b that when the number of Si atoms is greater than that of Al atoms, the structural content of Si-O-Al increases with the increase in Al_2_O_3_ concentration, and the maximum value is about 45%. When the number of Al atoms in the simulation system exceeds that of Si atoms, the possibility of forming Si-O-Al structure gradually decreases, Q^3^, Q^2^ and Q^1^ polymerize to form Q^4^ and Q^5^, and the possibility of forming Al-O-Al structure and O_t_ increases. The function of O_t_ is to balance the surplus negative charge of [AlO_4_]^5−^ tetrahedron structure. In O (Al, Al, Si) and O (Al, Al, Al) structures, the Al-O bond acts as a coordination bond to balance the charge distribution in the system. The structures of O (Al, Al, Al) can be divided into public side and non-public side structures according to their formation. In the case of the public side structure, two edges from two [AlO_4_]^5−^ tetrahedrons overlap, which promotes the release of O atoms. This type of structure formation leads to structural deformation and increased energy, thereby increasing structural instability. The structures of O (Al, Al, Si) and O (Al, Al, Al) are shown in Figure 12c.

**Figure 13:**
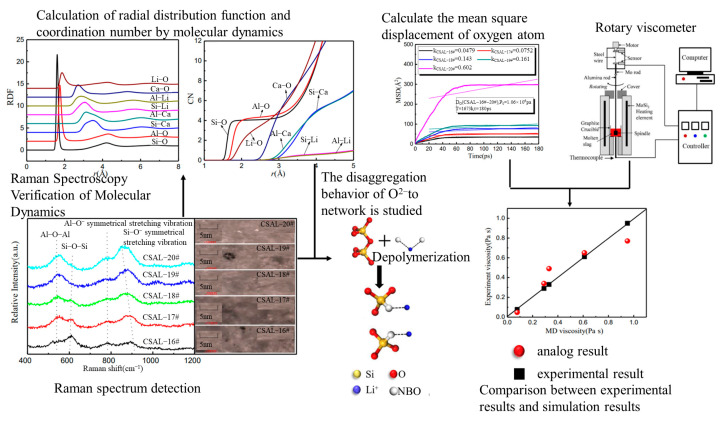
Combining molecular dynamics simulation results with experimental data [98].

Wang [102] took the CaO-SiO_2_-Al_2_O_3_-Li_2_O quaternary slag system as the research object, used molecular dynamics simulation to study its structural characteristics and viscosity, and used Raman spectroscopy detection methods and viscosity test experiments to verify the molecular dynamics simulation calculation results. According to molecular dynamics calculation, with the increase in Li_2_O, the bond-breaking effect of metal oxide Li_2_O on the Al-O bond is strengthened, and the average bond lengths of Al-Li and Al-Ca fluctuate obviously, the Li^+^ around tetrahedron [AlO_4_]^5−^ increases, and Li^+^ may replace Ca^2+^ to provide charge balance or charge compensation for tetrahedron [AlO_4_]^5−^. In the Raman spectrum detection, it can be seen that the increase in Li_2_O will lead to the transformation of the structural unit Al-O-Si to Al-O-Al, which can enhance the connectivity of the Al-O structure. This also shows that Li_2_O has a strong charge compensation ability for [AlO_4_]^5−^. The depolymerization effect of O^2−^ on the network mechanism was also studied by combining experiments and simulations. Under the action of high temperature, Li_2_O with high optical basicity and O^2−^ in SiO_2_ with low optical basicity will be redistributed, the Si-O-Si structure is depolymerized into Si-O^−^ structure, and the melt structure will be simplified. The mean square displacement obtained through molecular dynamics is processed, and the self-diffusion coefficient of the oxygen atom is obtained from the mean square displacement, and then the viscosity is obtained via the self-diffusion coefficient. By comparing the viscosity measured by the rotational viscometer, it can be seen that the simulation results and the experimental results are in good agreement, and the viscosity decreases with the increase in Li_2_O. A summary of the molecular dynamics simulation calculation results combined with the experiments is shown in Figure 13.

**Figure 14:**
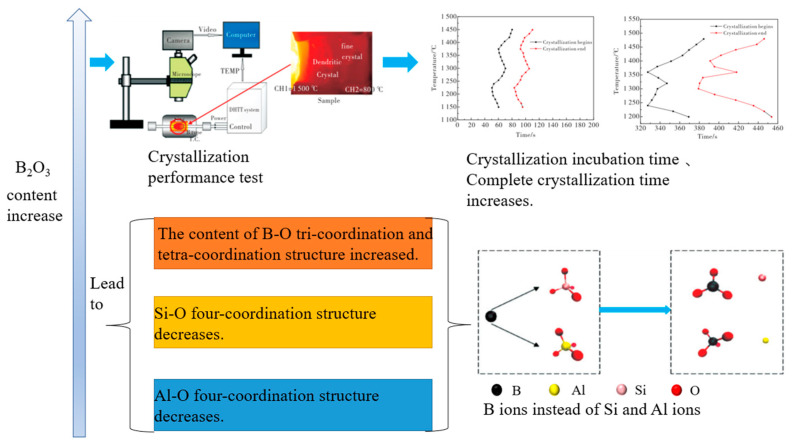
The effect of increasing B_2_O_3_ content on the structure.

Jiang et al. [103] carried out a molecular dynamics simulation on the basic structural system of blast furnace slag, SiO_2_-Al_2_O_3_-CaO-MgO quaternary slag system. Under the condition of different alkalinity, the mass ratio of MgO/Al_2_O_3_ was changed, and the structural unit and bond angle transmission characteristics were analyzed. Analysis of RDF shows that the MgO/Al_2_O_3_ ratio does not change the bond lengths of Si-O and Al-O significantly. In terms of CN_S_ analysis, the coordination numbers of Al-O and Si-O show an upward trend, and this indicates that the ratio of MgO/Al_2_O_3_ will weaken the stability of the system network structure. The MgO/Al_2_O_3_ ratio increases, the NBO concentration increases, and the BO concentration decreases, which can indicate that the increase in the ratio will lead to more Mg^2+^ damage to the network structure, resulting in the transition of bridging oxygen to non-bridging oxygen. Wu et al. [104] studied the effect of basicity on the slag system structure and viscosity characteristics in the quaternary slag system CaO-SiO_2_-Al_2_O_3_-FeO. The results show that with the increase in alkalinity, the silicon–oxygen and aluminum–oxygen networks depolymerize into simple structures and the diffusion coefficient of atoms increases. Increasing the content of Al_2_O_3_ causes the slag system to tend to form a more complex structure. Wang [105] studied the effect of B_2_O_3_ on the properties and microstructure of slag in the B_2_O_3_-CaO-Al_2_O_3_-SiO_2_ slag series. Studies have shown that B_2_O_3_ affects the viscosity and transition temperature of the slag system. When the B_2_O_3_ content is lower than 9%, the viscosity and transition temperature of the slag will decrease significantly with the increase of the B_2_O_3_ content. The crystallization and melting properties were studied using a DHTT-Ⅱ melting crystallization thermometer, and it was shown that the increase in B_2_O_3_ content led to the prolongation of crystallization incubation time and complete crystallization time. B-O is more stable than Al-O and Si-O coordination structures, and B ions in the system can replace Si and Al ions to form B-O three-coordination and four-coordination structures, the Si-O four-coordination structure and the Al-O four-coordination structure will decrease, and the structure of the slag will change from the framework structure of [SiO_4_] and [AlO_4_] to the layered structure of [BO_3_]. This change makes the structure more relaxed, resulting in a decrease in the degree of polymerization and viscosity of the slag. The effect of increasing B_2_O_3_ content on the structure is shown in Figure 14.

Shimoda et al. [106] studied the CaO-Al_2_O_3_-SiO_2_-MgO slag system and showed that with the increase in MgO content, SiO_4_ would depolymerize, viscosity would decrease, and the fluidity of slag would be improved. Mongalo et al. [107] studied the relationship between structural properties and electrical conductivity in CaO-MgO-Al_2_O_3_-SiO_2_ slag, and the ratio of NBO and BO was determined by simulation; the amount of NBO increased with increasing alkalinity. Zhao et al. [108] studied the effect of CaO/Al_2_O_3_ mass ratio on the structural properties of refining slag and analyzed the pairing distribution function, coordination number, microstructure unit, and diffusion capacity. The increase in the CaO/Al_2_O_3_ ratio did not affect the bond lengths of Si-O, Al-O, Ca-O, Mg-O, and did not affect the bond angles of O-Si-O. The higher the ratio of CaO/Al_2_O_3_, the more Al^3+^ in the slag as a network former, which promoted the transformation of FO and NBO to TO and BO and improved the polymerization degree of the system. The diffusivity of different atoms was Mg > Ca > O > Al > Si.

To sum up, most of the ensembles selected by the above scholars are NVT ensembles, which keep the particle number, volume, and temperature constant. Shimoda chooses the ensemble as the NPT ensemble, and the particle number N, the pressure P and the temperature T are constant; that is, the isothermal and isobaric ensemble. The use of the potential function is also different; there is BMH potential, CMAS94 potential, Lennard–Jones potential, and Morse potential. The research of molecular dynamics in the aluminosilicate system is mainly to study its structure through radial distribution function, bond length, bond angle distribution and coordination number. Not only can the molecular dynamics predict the complex quaternary slag system structure, but it can also predict its dynamic properties and establish the relationship between structural properties and viscosity. However, in the actual production, not all of them are pure aluminosilicate slag systems, and there will inevitably be other trace impurities. For example, in the research of special steel, trace impurities will also have a significant impact on the structure. Therefore, other elements that will exist should be added to the research on slag systems in combination with the actual situation. In addition, most of the above studies are on the slag element mass ratio and element-to-slag system structure, which is less related to temperature. It is necessary to further study the change of temperature and the influence of heating and cooling rates on the structure, as it will play an important role in the study of complex slag system and the explanation of its reaction mechanism. Finally, the research of molecular dynamics in aluminosilicate and the selection of potential function are summarized in Table 5. According to Table 5, researchers have mostly studied the structure and viscosity of aluminosilicate slag systems and adopted a variety of potential functions, among which Morse’s potential function can describe resonance phenomena, such as molecular tensile vibration. In addition, Morse potential function also considers the variation of vibration frequency with amplitude.

From the above analysis, it can be found that there are few studies on the combination of molecular dynamics simulation and laboratory experiments, and the close combination of the two will provide more accurate and reliable results for molecular dynamics simulation. By combining experimental data with simulation technology, researchers can deeply study and understand the mechanism of intermolecular interaction, particle motion and chemical reaction. Experimental data provide true and reliable observation results, and through molecular dynamics simulation, researchers can simulate and explore a large number of molecular systems to explore their behavior and properties. The experimental results are compared with the simulation data via the verification method, so as to verify the accuracy and applicability of the simulation. This comprehensive method can not only help researchers identify and correct potential simulation errors, but also provide a more comprehensive understanding and explanation of the behavior of molecular systems.

Therefore, researchers should actively advocate and adopt the method of combining verification to closely combine experiments with molecular dynamics simulation to improve the depth and accuracy of research. This comprehensive method will create new possibilities for scientific research and technological development and promote progress and innovation in the field of molecular dynamics.

## 4. Conclusions and Outlook

In summary, domestic and foreign scholars have carried out molecular dynamics simulation studies on different slag systems and obtained some conclusions that are of great significance to the microstructure. Based on the above research, the ensemble adopted by most scholars is the NVT (canonical) ensemble. In the simple slag system, the BMH potential function is mostly selected; in the complex slag system, there are many kinds of potential functions selected. Different potential functions are used to study different particles so that more accurate results can be obtained. However, according to the above analysis, the simulation mainly includes research on the basic structure of slag and the diffusivity of atoms. There is slightly less research on temperature, and more in-depth research should be carried out, such as an investigation of the influence of different heating and cooling rates on the microstructure, and the simulation conditions are too ideal, so trace impurities in the slag, such as Cu, Pb and S, should be considered. The effect of small amounts of impurities on the structure is huge, and molecular dynamics are used to investigate this more deeply. Because of the above-mentioned molecular dynamics research in different slag systems, this paper has the following prospects:

1. In the future, researchers will devote themselves to developing more targeted new potential functions to describe the behavior and characteristics of microstructures more accurately. This new potential function is used to solve problems in small-scale systems such as atoms and molecular clusters. There is a need for in-depth study and analysis of microstructure, design of more accurate and reliable models, and promotion of the research and application at atomic and molecular levels.
2. In the future, efforts will be made to further integrate molecular dynamics simulation methods with experiments to jointly explore the viscosity and other macroscopic properties of slag systems, such as activity, thermodynamic properties, and oxidation. Through accurate simulation and experimental verification, the complex behavior and interaction of slag systems can be revealed, and the understanding of microstructure in slag can be improved.
3. Most molecular dynamics simulation environments are usually assumed to be vacuum environments because vacuum environments can reduce the influence of intermolecular interaction and make the simulation simpler and faster. However, under the actual laboratory conditions, it is difficult to achieve a complete vacuum environment. Laboratories often choose to use inert gases, such as argon, and future research and technical development may create more accurate molecular dynamics simulations in different gas environments. By simulating the behavior of samples in different gas environments, the actual situation in the laboratory can be more accurately reflected, and the reliability and accuracy of the simulation results can be increased.
4. In the phosphate slag system, important processes such as crystal growth and phase transformation are usually complex and long. These processes involve a series of microscopic changes such as interface reconstruction and energy redistribution. In future research, the simulation time of the phosphate slag system can be further increased to obtain a more comprehensive and detailed behavior evolution. By prolonging the simulation time, researchers observe the more complex and slow process, gain deeper insight, and understand the evolution process of the system.

## Figures and Tables

**Table 1:** Molecular dynamics common algorithms.

Algorithm	Advantage	Disadvantage	References
Verlet algorithm	The calculation process is simple and easy to apply	It is not a self-starting algorithm, and its accuracy is inaccurate	[31]
Speed Verlet algorithm	The position, velocity and acceleration are given at the same time without reducing the accuracy	The calculation process is complicated	[32]
Leapfrog algorithm	With an explicit velocity term, there is no need to calculate the difference between two larger quantities 2r(t) and r(t − δ)	The position and speed cannot be synchronized	[33]
Beeman algorithm	Can maintain energy conservation	The amount of calculation is large	[34]
Rahman algorithm	Obtains a more accurate solution	Time consuming with a large amount of calculation	[35]

**Table 2:** Research on phosphate and selection of potential function.

Research System	Research Content	Potential Function	Ensemble	References
CaO-P_2_O_5_/Na_2_O-P_2_O_5_	Structural properties	BMH	NVT	[80]
CaO-SiO_2_-P_2_O_5_	Organizational characteristics of the structure	BMH	NVT	[83]
2ZnO·P_2_O_5_-2Na_2_O·P_2_O_5_	Structural properties	BMH	—	[84]
P_2_O_5_-Fe_2_O_3_-FeO-Na_2_O	the effect of the addition of Na_2_O on the structure	Buckingham + Stillinger–Weber	NVT	[85]
Li_2_O–P_2_O_5_–LiCl	The effect of the addition of LiCl on the structure	BMH	—	[86]
P_2_O_5_-CaO-Na_2_O	Study the structure of phosphate glass	Buckingham + Born-Mayer	NVT	[87]

Note: — is not stated in the text.

**Table 3:** Research on silicate slag system and selection of potential function.

Research System	Research Content	Potential Function	Ensemble	References
CaO-SiO_2_	Melt structure, viscosity, conductivity	BMH	NVT	[88]
CaO-SiO_2_-CaF_2_	Effect of CaF_2_ on slag structure	BMH	—	[89]
CaO-SiO_2_-CaF_2_	Melt structure	BMH + Buckingham + MiTra	—	[90]
CaO-SiO_2_-CaF_2_	Melt structure property	BMH	—	[91]
CaO-P_2_O_5_-SiO_2_	Melt structure	BMH	NVT	[92]
CaO-SiO_2_-P2O_5_-FeO	Melt structure and viscosity	BMH	NVT	[93]
CaO-B_2_O_3_-SiO_2_-TiO_2_	Melt structure	BMH	—	[94]

Note: — is not stated in the text.

**Table 4:** Research on aluminate slag system and selection of potential function.

Research System	Research Content	Potential Function	Ensemble	References
SiO_2_-Al_2_O_3_	Melt structure	BMH	NVT	[95]
CaO-Al_2_O_3_	Microstructure unit distribution, viscosity, conductivity	BMH	—	[96]
CaO-Al_2_O_3_-Na_2_O	Structural characteristics of slag	Buckingham	NVT	[97]
CaO-Al_2_O_3_-CaF_2_	Melt structure and viscosity	Buckingham	—	[98]

Note: — is not stated in the text.

**Table 5:** Research on aluminosilicate slag system and selection of potential function.

Research System	Research Content	Potential Function	Ensemble	References
CaO-Al_2_O_3_-SiO_2_	Electrical conductivity, structure	BMH	—	[100]
CaO-Al_2_O_3_-SiO_2_	Melt structure and viscosity	BMH	NVT	[101]
CaO-SiO_2_-Al_2_O_3_-Li_2_O	Melt structure	BMH	NVT	[102]
SiO_2_-Al_2_O_3_-CaO-MgO	Slag structure	CMAS94	NVT	[103]
CaO-SiO_2_-Al_2_O_3_-FeO	Structure and viscosity	Lennard-Jones	NVT	[104]
B_2_O_3_-CaO-Al_2_O_3_-SiO_2_	Structure and viscosity	BMH	NVT	[105]
CaO-Al_2_O_3_-SiO_2_-MgO	Structure and viscosity	Morse + BMH	NPT	[106]
CaO-MgO-Al_2_O_3_-SiO_2_	Structure, electrical conductivity	Morse	NVT	[107]
SiO_2_- Al_2_O_3_- CaO-MgO	Structure	BMH	NVT	[108]

Note: — is not stated in the text.

## Data Availability

Not applicable.
